# Supplementary material for: Consent is a confounding factor in a prospective observational study of critically ill elderly patients
Source: PLoS One. 2022 Oct 27;17(10):e0276386. doi: 10.1371/journal.pone.0276386 (PMC9612504; doi:10.1371/journal.pone.0276386)
Supplement: S1 Table — (DOCX) [file pone.0276386.s001.docx]

**Supporting table 1a sensitivity analysis with England included in no consent group.**

| **UNADJUSTED** |  |  |  |
| --- | --- | --- | --- |
|  | HR consent vs no consent | 95% CI | P-value |
| Overall survival | 0.69 | 0.63-0.75 | <0.0001 |
| **ADJUSTED Cox ^1^** |  |  |  |
|  | HR consent vs no consent | 95% CI | P-value |
| Overall survival | 0.72 | 0.66-0.80 | 0.0001 |
|  |  |  |  |
| **IPW weighted Cox model ^1^** |  |  |  |
|  | HR consent vs no consent | 95% CI | P-value |
| Overall survival | 0.74 | 0.68-0.82 | 0.0001 |
|  |  |  |  |
| **Propensity score weighting ^1^** | HR consent vs no consent | 95% CI | P-value |
| Overall survival | 0.74 | 0.67-0.81 | 0.0001 |
| Mechanical ventilation | 0.96 | 0.88-1.1 | 0.68 |
| Vasoactive drugs | 0.79 | 0.72-0.85 | <0.0001 |
| Renal replacement | 0.83 | 0.68-1.01 | 0.0627 |
| Limitation of care | 1.02 | 0.92-1.1 | 0.68 |

^1^ including variables age, gender, habitat, frailty, reason for ICU admission and SOFA score

**Supporting table 1 b Sensitivity analysis with England included in consent group**

| **UNADJUSTED** |  |  |  |
| --- | --- | --- | --- |
|  | HR consent vs no consent | 95% CI | P-value |
| Overall survival | 0.72 | 0.66-0.79 | <0.0001 |
| **ADJUSTED Cox ^1^** |  |  |  |
|  | HR consent vs no consent | 95% CI | P-value |
| Overall survival | 0.95 | 0,87-1.06 | 0.30823 |
|  |  |  |  |
| **IPW weighted Cox model ^1^** |  |  |  |
|  | HR consent vs no consent | 95% CI | P-value |
| Overall survival | 0,95 | 0.87-1.06 | 0.429 |
|  |  |  |  |
| **Propensity score weighting ^1^** | HR consent vs no consent | 95% CI | P-value |
| Overall survival | 0.92 | 0.83-1.02 | 0.107 |
| Mechanical ventilation | 0.8 | 0.72-0.88 | <0.0001 |
| Vasoactive drugs | 0.73 | 0.66-0.80 | <0.0001 |
| Renal replacement | 0.94 | 0.76-1.16 | 0.57 |
| Limitation of care | 1.06 | 0.93-1.2 | 0.351 |

^1^ including variables age, gender, habitat, frailty, reason for ICU admission and SOFA score
