# Supplementary material for: Consent is a confounding factor in a prospective observational study of critically ill elderly patients
Source: PLoS One. 2022 Oct 27;17(10):e0276386. doi: 10.1371/journal.pone.0276386 (PMC9612504; doi:10.1371/journal.pone.0276386)
Supplement: Attachment — Submitted filename: Review_2022-08_12.pdf [file pone.0276386.s004.pdf]

This manuscript describes a secondary data analysis study for a prospective, observational study of the acutely admitted very old intensive care patients (the VIP2 study). The findings in the original VIP2 study (conducted in 22 European countries) was recently published. In this secondary data analysis study, the authors identified the methods for Information Consent as a major confounding factor in prospective observational studies of critically ill elderly patients. The finding is interesting and potentially useful for readers. The statistical analysis methods are relatively simple but reasonable.

However, I have some concerns:

Major concerns:

1. In table 1, some numbers seem problematic, for example, the last three row:

SOFA median (95% CI)	8 (8-9)	6 (6-7)
CFS[a] median (95% CI)	4 (4-5)	4 (4-5)
IQCODE[b] median (95% CI)	3.31 (3.31-3.38)	3.19 (3.19-3.25)

How could the lower bound of the 95% confidence interval (i.e. the 2.5% quantile) be the same as the sample median (50% quantile)? Although this is potentially possible under some rare scenarios, it is more likely due to some careless mistakes in data analysis. If the authors do think the current results are correct, please show the histogram of these variables, in the response letter, so that reviewers can confirm the results.

2. The references in the revised manuscript seem to be misplaced. For example, reference [3] in the main text seem to be the wrong reference (it should be the reference [4] in the reference list. In addition there 9 references in the reference list, only 8 were referred in the main text.
3. It was mentioned in the last sentence of the Result section that "*In the regression analysis consent or no consent was an independent variable in addition to SOFA score and IQCODE.*" However, the results of the regression analysis were not presented anywhere (as either table or figure) in the manuscript.

Minor concerns:

4. Please spell out which analysis were used to calculate the p value in Figure 1.
5. The first sentence of the Introduction, the parentheses do not match.
"*In a recently published, prospective, observational study of the acutely admitted very old intensive care patients (⩾80 years old), the VIP2 study) conducted in 22 European countries, we found an overall 30-day survival of 61%. Factors predicting mortality were frailty at admission, ICU admission categories and degree of organ failure at admission [1].*"
