# Supplementary material for: Consent is a confounding factor in a prospective observational study of critically ill elderly patients
Source: PLoS One. 2022 Oct 27;17(10):e0276386. doi: 10.1371/journal.pone.0276386 (PMC9612504; doi:10.1371/journal.pone.0276386)
Supplement: Attachment — Submitted filename: Response to reviewers final.doc [file pone.0276386.s003.doc]

5. Review Comments to the Author

Please use the space provided to explain your answers to the questions above. You may also include additional comments for the author, including concerns about dual publication, research ethics, or publication ethics. (Please upload your review as an attachment if it exceeds 20,000 characters)

Reviewer #1: Current paper presented an observational study comparing ICU length of stay (LOS), use of intensive care procedures for organ support and 30-day survival. The paper is written in hard to read English with sentences possibly grammatically correct but should be revised by a native speaker for easy reading. In the next section I am primarily focusing on the statistical and data analysis aspect of the project.

Response: One of the authors (S.Leaver) is a clinician and researcher living and working in London UK and has commented on the English writing (also in the first manuscript)

1. The paper gives very little information about the observational data to be analyzed. Table 1 presents some data but that is only the tip of the iceberg. Please give detailed demographic and clinical data as well as their standard deviation (when applicable).

For some variable mean and for some median is reported without reasoning. For observational data analysis detailed description of the data is must, so that a reader can understand what is being analyzed.

Response: We agree that too few clinical data were given, but we have given confidence interval of relevant data. We have as your request all clinical data in a new table 1 included all relevant clinical data now analyzed and discussed in the paper, we also have given mean values with 95% confidence interval or as % in counts.

Continuous variables are summarized as median (range) (IQR) and categorical as N(%).

2. The statistical methods used for analysis are either cryptic or without sufficient description. It seems only two group comparisons are made without addressing inherent bias (typical in any observational data) in the data. A propensity score or similar methods should have been used instead.

Response: We changed the analysis and detailed new analysis in the manuscript.

We followed reviewer's suggestion and used propensity score weighting to compare outcome between groups. We also added a curve showing survival of the two groups after adjustement for main patients characteristics.

«*The crude overall survival up to 90 days after ICU admission was estimated by the Kaplan-Meier method and compared between groups using a log-rank test.*

*Incidence of organ support and treatment limitations were estimated using cumulative incidence analysis considering ICU death and ICU discharge as competing risks. Univariate comparisons were performed using Gray’s test.*

*In order to adjust comparison of outcomes for patients characteristics Cox model were used for survival and cause-specific Cox models were used for organ support and treatment limitations.The following factors were used for adjustement: age, sex, SOFA, reason for ICU admission, frailty and habitat.*

*Adjusted survival curves were produced using an Inverse Probability Weighted (IPW) Kaplan-Meier estimation. Significance was tested using a Cox regression model weighted by the same weights (inverse probability-weighted Cox).*

*We then used then propensity score (PS) weighting to control for imbalances on observed variables between groups for all outcomes. The PS model included the same covariates namely: age, sex, SOFA, reason for ICU admission, frailty and habitat. Generalized boosted regression were used to estimate the propensity score and cases were then weighted to estimate the average effect of consent on the population. All tests are two-sided. The type-I error rate is fixed at 0.05. Analyses were performed using the R statistical software version 3.4.2 (R Foundation for Statistical Computing, Vienna, Austria [*[*https://www.R-project.org/*](https://www.R-project.org/)*]); IPW analysis were performed using the ipwpoint function of the IPW package. PS analysis were performed using the ps function of the Twang package and weighted analyses used the survey package.*»

3. How the figure 1 is produced is not clear. The paper also mentions “sensitivity analysis” and “regression analysis” in passing without going into detail about the results or how it is being carried out.

Response: We agree that the figure and its interpretation is insufficient, and have removed it from the recent version.

In short, the paper's statistical methods and its interpretation as well as presentation is below the publication standard. It is highly recommended to include a trained statistician to curate these areas.

Response:

We now hope the statistical analysis is improved sufficiently to meet the journal standards, and now even more underline the importance of not revealing type of consent in the methods of observational multicenter studies. A statistician has been used also for this last version (A. Boumendil).

Reviewer #2: This is and interesting and original paper on a very important topic: the consent to participate to clinical studies.
The Authors show that the lack of consent is related to scientific biases and to poor outcomes in the older sample of patients of this research.
The paper is well written and the English form is correct.
Data are well expressed and the results are exposed in a clear form. The discussion of results is very clear.

Response: We thank the reviewer for these positive remarks and underlining the importance of our main message.

Reviewer #3: The authors should be congratulated because the issue they address in this paper is methodologically relevant. The manuscript deals with analysis of potential selection bias and, more than relevant for the VIP2 study, the issue is relevant in general for clinical research in the acute medicine setting.
I have some issues that can improve the quality of the manuscript and the knowledge on the issue:
- please describe cause of admission for the two groups (A vs B)
- please describe the components of the SOFA score in the two groups (A and B)

Response:

Thank you for this comment, we have now included a table that reveals admittance categories for both cohorts (new table 2), and it reveals some interesting differences in that pure respiratory failure were more frequent (27.4% vs 21%) in group b (consent) while the more severe group with combined respiratory and circulatory failure at admittance were more frequent in group A (16.7 vs 9.5%)

We have now added a description of SOFA components for the two groups
